# A Proposition

**Published:** 1862-03

**Authors:** J. Taft


### A PROPOSITION.

We have made an arrangement with the author of "*The Dentist's Memorandum and Book of Engagements,*" by which we will for a short time furnish the Register for one year and a copy of the above work for 1862 at $3.00.

Those who wish to comply with this proposition will please send immediately, as we have but a limited number of copies of the work. It is one of the best works of the kind. The retail price is $1.00. J. Taft.

